# Supplementary material for: Hybrid Models and Biological Model Reduction with PyDSTool
Source: PLoS Comput Biol. 2012 Aug 9;8(8):e1002628. doi: 10.1371/journal.pcbi.1002628 (PMC3415397; doi:10.1371/journal.pcbi.1002628)
Supplement: Text S4 — Complete source code for the PyDSTool package (version 0.88.120504). Includes API documentation and help files linking to web pages. This file is identical to the current public release on Sourceforge.net. (ZIP) [file pcbi.1002628.s004.zip › PyDSTool/html/PyDSTool.Toolbox.optimizers.tests.test_powell.Powell-class.html]

xml version="1.0" encoding="ascii"?


PyDSTool.Toolbox.optimizers.tests.test\_powell.Powell


| Home | Trees | Indices | Help | PyDSTool |
| --- | --- | --- | --- | --- |

Package PyDSTool ::
Package Toolbox ::
Package optimizers ::
Package tests ::
Module test\_powell ::
Class Powell

[hide private]

[frames] | no frames]

# Class Powell

source code

The Powell function


|  |  |
| --- | --- |
| Instance Methods | [hide private] |

|  |  |
| --- | --- |
| \_\_call\_\_(self, x)  Get the value of the Powell function at a specific point | source code |

|  |  |
| --- | --- |
| gradient(self, x)  Evaluates the gradient of the function | source code |

|  |  |
| --- | --- |
| hessian(self, x)  Evaluates the gradient of the function | source code |

| Home | Trees | Indices | Help | PyDSTool |
| --- | --- | --- | --- | --- |

|  |  |
| --- | --- |
| Generated by Epydoc 3.0.1 on Fri May 4 15:24:09 2012 | http://epydoc.sourceforge.net |
